# Impact of the first COVID-19 outbreak on mental health service utilisation at a Dutch mental health centre: retrospective observational study

**DOI:** 10.1192/bjo.2021.1049

**Published:** 2021-11-17

**Authors:** Man Wei Chow, Eric O. Noorthoorn, André I. Wierdsma, Marte van der Horst, Nini de Boer, Sinan Guloksuz, Jurjen J. Luykx

**Affiliations:** Department of Psychiatry, UMC Utrecht Brain Centre, University Medical Centre Utrecht, Utrecht University, The Netherlands; and Department of Translational Neuroscience, UMC Utrecht Brain Centre, University Medical Centre Utrecht, Utrecht University, The Netherlands; Department Training of Psychiatrists, GGNet Mental Health, The Netherlands; Department of Psychiatry, Erasmus Medical Centre, The Netherlands; Department of Psychiatry, UMC Utrecht Brain Centre, University Medical Centre Utrecht, Utrecht University, The Netherlands; Department of Translational Neuroscience, UMC Utrecht Brain Centre, University Medical Centre Utrecht, Utrecht University, The Netherlands; and Department Training of Psychiatrists, GGNet Mental Health, The Netherlands; Department of Psychiatry, UMC Utrecht Brain Centre, University Medical Centre Utrecht, Utrecht University, The Netherlands; and Department of Translational Neuroscience, UMC Utrecht Brain Centre, University Medical Centre Utrecht, Utrecht University, The Netherlands; Department of Psychiatry and Neuropsychology, School for Mental Health and Neuroscience, Maastricht University Medical Centre, The Netherlands; and Department of Psychiatry, Yale School of Medicine, Connecticut, USA; Department of Psychiatry, UMC Utrecht Brain Centre, University Medical Centre Utrecht, Utrecht University, The Netherlands; Department of Translational Neuroscience, UMC Utrecht Brain Centre, University Medical Centre Utrecht, Utrecht University, The Netherlands; and Department Training of Psychiatrists, GGNet Mental Health, The Netherlands

**Keywords:** Psychotic disorders, mental health service utilisation, COVID-19, telepsychiatry, face-to-face out-patient contact

## Abstract

**Background:**

Previous studies into mental health service utilisation during the COVID-19 pandemic are limited to a few countries or specific type of service. In addition, data on changes in telepsychiatry are currently lacking.

**Aims:**

We aimed to investigate whether the COVID-19 pandemic is associated with changes in mental health service utilisation, including telepsychiatry, and how these changes were distributed among patients with mental illness during the first COVID-19 outbreak.

**Method:**

This retrospective study obtained routinely assessed healthcare data from a large Dutch mental healthcare institute. Data from the second quarter of 2020 (the first COVID-19 outbreak period) were compared with the pre-pandemic period between January 2018 and March 2020. Time-series analyses were performed with the quasi-Poisson generalised linear model, to examine the effect of the COVID-19 lockdown and the overall trend of mental health service utilisation per communication modality and diagnostic category.

**Results:**

We analysed 204 808 care contacts of 28 038 patients. The overall number of care contacts in the second quarter of 2020 remained the same as in the previous 2 years, because the number of video consultations significantly increased (B = 2.17, *P* = 0.488 × 10^−3^) as the number of face-to-face out-patient contacts significantly decreased (B = −0.98, *P* = 0.011). This was true for all different diagnostic categories, although this change was less pronounced in patients with psychotic disorders.

**Conclusions:**

Diminished face-to-face out-patient contacts were well-compensated by the substantial increase of video consultations during the first COVID-19 outbreak in The Netherlands. This increase was less pronounced for psychotic disorders. Further research should elucidate the need for disorder-specific digital mental healthcare delivery.

The COVID-19 pandemic, declared by the World Health Organization as a global health crisis on 11 March 2020,^1^ has affected countries worldwide in various ways. During the first COVID-19 outbreak in The Netherlands, from late March to June 2020, more than 10 000 individuals died from (suspected) COVID-19 infection (i.e., 58.1 deaths per 100 000 individuals).^2^ Restrictions implemented by the Dutch Government to prevent the virus from spreading included temporarily limiting access to schools, public transport, restaurants and bars, and self-isolation as much as possible.^3^ As a result of the pandemic and social distancing, the physical and mental well-being of the general population have been significantly affected.^4–6^ People with mental illness are likely to be affected disproportionally compared with the general population, because of higher chances of somatic and psychosocial inequalities.^7^ Subsequently, increasing pressure on mental health services raises concerns about service capacity and the ability to maintain good quality-of-care provision.^8^ Furthermore, a study performed in China hints at regional differences in mental health service utilisation contingent on the severity of the COVID-19 outbreak: in heavily affected regions, residents experienced the most psychological distress and therefore required more mental healthcare.^9^ Several studies have examined the association between the COVID-19 pandemic and mental health service utilisation across the full range of psychiatric disorders. Two studies found differences between patients with mental illness: referrals and admissions to secondary mental health services during the first outbreak in the UK decreased less for patients with severe mental illness than for patients without severe mental illness,^10^ whereas the second study found elevated numbers of admissions for patients with psychotic and bipolar disorders.^11^ Moreover, another British study examining the effects of lockdown on referrals to mental health services during the first COVID-19 outbreak detected an increase in referrals to mental health emergency services after an initial drop in referrals at the beginning of the outbreak.^12^ The same trend in referrals was also observed for those with severe mental illness, pre-existing depression and anxiety.^12^ Finally, during the lockdown in India, patients with schizophrenia presented relatively often at emergency services compared with before the COVID-19 pandemic.^13^

To our knowledge, several studies to date have examined the quantitative changes in mental health service use among patients with pre-existing mental illnesses during the pandemic. However, no study has examined the discrepancies in telepsychiatry use among subgroups with different mental illnesses, even as mental health systems worldwide have shifted to more telepsychiatry-integrated care since the COVID-19 pandemic.^14–16^ Knowledge about such changes in other countries may elucidate the global versus regional effects of the pandemic on mental health services, as well as uncover the potential of alternative mental healthcare opportunities that may be implemented, e.g. with regard to telepsychiatry.^17^ In light of these knowledge gaps, we aimed to examine associations between the COVID-19 pandemic and mental health service utilisation, including analyses of telepsychiatry utilisation, among patients with mental illness. Our objectives were to examine (a) changes in the number of care contacts for several communication modalities by comparing data from the second quarter of 2020, which corresponded to the first COVID-19 outbreak in The Netherlands, with the pre-pandemic mental healthcare utilisation period between January 2018 and March 2020; and (b) changes in the number of care contacts for several diagnostic categories when comparing the corresponding periods. We expected a decrease in face-to-face contacts inversely proportional to an increase in telepsychiatry contacts. Because of functional and cognitive impairments, we also expected a smaller decrease in face-to-face contacts and smaller increase in telepsychiatry contacts for patients with psychotic disorders compared with patients with other psychiatric disorders.

## Method

### Study design and participants

In this retrospective, observational cohort study, data of out-patients were collected from the mental healthcare records of a large Dutch mental healthcare institute, GGNet. This institute delivers both primary and secondary mental healthcare in Gelderland, the largest province of The Netherlands. Its catchment area has around 2 million inhabitants and includes several cities as well as more rural areas, which reflects the general geography of The Netherlands fairly well. All electronic healthcare records from January 2018 until June 2020 were examined. We specifically compared the data from the second quarter of 2020, which corresponded to the first COVID-19 outbreak period in The Netherlands (April, May and June), with the data from between January 2018 and March 2020. We included all patients with a clinician-based diagnosis of mental illness according to the DSM-5 criteria.^18^ We excluded patients with a neurocognitive disorder as a primary diagnosis, given that the majority of specialised care to this group is delivered by facilities outside of GGNet. The procedures in this study were subjected to ethical assessment by the Ethics Committee of the University of Twente in Enschede. This committee deemed that the protocol was not subject to the requirements of the Medical Research (Human Subjects) Act, as they were performed on anonymous data.

### Data collection

Data were obtained from anonymised electronic medical records at the GGNet Mental Health Trust. The database consisted of routinely assessed mental healthcare records on all care contacts of patients with healthcare professionals from January 2018 to June 2020. In March 2020, all healthcare workers at GGNet were instructed to continue carrying out care as needed, meaning that if face-to-face contacts were needed, these should be scheduled, but if video consultations or telephone contacts were possible, these could be viable alternatives. As no further instructions were given, this allowed us to investigate in a fairly naturalistic setting how mental healthcare would change during the pandemic. Variables such as gender, marital status or educational level were excluded from the database to avoid traceability of patients, in line with Dutch privacy regulations.

### Variables

Mental health service utilisation was defined as the frequency of care contacts with health professionals. We considered changes in the number of (several types of) care contacts and changes in the number of care contacts per diagnostic category as outcomes to be investigated for their association with the first COVID-19 outbreak. Care contacts were categorised by main diagnostic categories and communication modalities. The following variables were extracted:

- Age category: youth (<18 years old), adult (18–64 years old) and elderly (≥65 years old).
- Main diagnostic categories: we categorised participants according to the main psychiatric diagnosis, with the main referring to the most important diagnosis as indicated by the treating clinician. The main DSM-5 diagnoses of interest included all the following diagnoses as listed in their respective sections of the DSM-5: anxiety disorders, depressive disorders, trauma- and stressor-related disorders, schizophrenia spectrum and other psychotic disorders, bipolar disorders, developmental disorders, eating disorders, personality disorders, substance use disorders and patients for whom diagnosis was intentionally delayed. However, we need to keep in mind that substance use disorders in The Netherlands are often not treated at general mental healthcare centres, but rather at centres specialised in addiction care services. Substance use disorder as a primary diagnosis occurrence in the current database is scant (*n* = 409, 0.2% of the patients; Table 1). Obsessive–compulsive disorders were grouped under anxiety disorders and paraphilic disorders were grouped under personality disorders. ‘Not yet diagnosed’ patients were patients that had not yet received a diagnostic classification but had one or more emergency contacts. To adhere to clinical practice in psychiatry where delaying diagnosis until later stages is commonplace, we included these patients in our analyses. In addition, the anxiety–depression–post-traumatic stress disorder (ADP) cluster was used to refer to anxiety disorders, obsessive–compulsive disorders, depressive disorders, and trauma- and stressor-related disorders, given their shared neurobiological underpinnings.^19^ Moreover, the ADP cluster is used as a reference group to compare outcomes on mental health service use with other diagnostic categories, including those with severe mental illness. Schizophrenia spectrum and other psychotic disorders were clustered as psychotic disorders.
- Communication modalities: video consultations, telephone contacts, face-to-face out-patient contacts and home visits.

**Table 1:** Descriptive statistics of the number of care contacts per variable in the second quarter of each year and from January 2018 to June 2020

Variables	Quarter 2, 2018, *n* = 68 991	Quarter 2, 2019, *n* = 66 387	Quarter 2, 2020, *n* = 69 430	January 2018 to June 2020, *N* = 204 808
	%	%	%	%
Mean age in years (±s.d.)	43.4 (±19.0)	42.3 (±18.7)	41.6 (±18.6)	42.3 (±18.8)
Youth (<18 years old)	8.7	7.5	7.6	7.9
Adults (18–64 years old)	76.6	78.4	78.8	77.8
Elderly (≥65 years old)	14.7	14.2	13.7	14.3
Diagnostic categories^a^				
ADP cluster	35.2	37.1	36.0	36.3
Psychotic disorders	13.2	13.6	12.3	13.3
Bipolar disorders	6.2	6.4	5.4	6.2
Developmental disorders	10.0	10.7	10.0	10.3
Eating disorders	2.9	3.4	3.3	3.1
Personality disorders	14.4	14.6	15.4	14.8
Substance use disorders	0.5	0.1	0.1	0.2
Not yet diagnosed	15.7	12.1	15.5	13.8
Communication modalities^a^				
Video consultations	0.0	0.0	13.1	1.5
Telephone contacts	15.8	14.5	32.0	17.9
Face-to-face out-patient contacts	67.4	68.8	39.3	63.7
Home visits	12.6	10.2	6.1	10.6

ADP, anxiety–depression–post-traumatic stress disorder.

a. The proportion (%) of all psychiatric diagnoses and communication modalities does not add up to 100% because diagnoses with few observations and administrative workload related to indirect patient care were omitted, respectively.

### Statistical analysis

Descriptive statistics were used to summarise patient and care contact characteristics comparing the second quarter of 2020, when COVID-19 measures were introduced, with the same quarters of 2018 and 2019.

To evaluate the effect of COVID-19 measures on the number of contacts with mental health services, we analysed time-series data with the R statistical package (version 3.6.2).^20^ Mental healthcare contacts were aggregated per month in total and stratified by type of communication modality and diagnostic category. To model changes in the number of care contacts per month, we used quasi-Poisson generalised linear model with a log-link function and number of contacts 3 months earlier to account for autocorrelation. All models included fixed covariates for the implementation of the COVID-19 measures and time to capture the effect of the COVID-19 lockdown and overall trend. Models including an interaction term of the second quarter of 2020 and types of communication modality or diagnostic category were tested to explore differences in mental health service utilisation associated with the COVID-19 measures. Model selection was based on the Wald test, with alpha set at 5%.

## Results

A total number of 204 808 care contacts for 28 038 patients with mental illness were included in our analyses (Table 1). For the second quarters of 2018 and 2019, the mean age of the patients was 42.3 years, with ages ranging from 23.49 to 61.19 years (s.d. ±18.85), and for the second quarter of 2020, the mean age was 41.61 years, with ages ranging from 23.06 to 60.16 years (s.d. ±18.55) (Table 1). The percentages of the total number of care contacts per psychiatric diagnosis in the second quarters of 2018 and 2019 and the second quarter of 2020 remained fairly stable over all diagnostic categories.

**Fig. 1:**
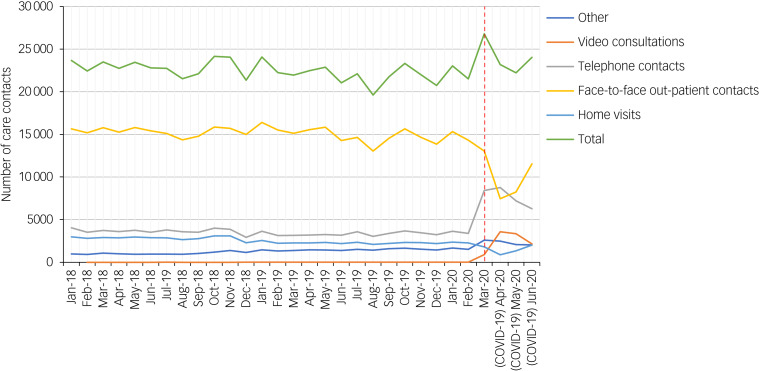
Number of care contacts by communication modality per month, from January 2018 to June 2020. The day of lockdown announcement in The Netherlands, as of 16 March 2020 (red dashed line). Video consultations (orange line) show a substantial increase, whereas face-to-face out-patient contacts (yellow line) show a significant decrease after the declaration of lockdown. ‘Other’ denotes to administrative activities associated with indirect patient care.

In the second quarter of 2020, the absolute total number of care contacts was elevated, as illustrated by the spike on the green line in Figure 1, but there was no significant change in the overall number of care contacts: since January 2018, the frequency of care contacts per month has been fluctuating between 20 000 and 25 000 (B = 2.92 × 10^−2^, *P* = 0.584; Fig. 1). However, there were changes observed in the level of communication modality. The number of video consultations increased substantially in the second quarter of 2020 compared with the pre-pandemic trend, where video consultations were hardly used (B = 2.17, *P* = 0.488 × 10^−3^; Fig. 1). On the other hand, the number of face-to-face out-patient contacts decreased significantly in the second quarter of 2020 (B = −0.98, *P* = 0.011; Fig. 1). Moreover, home visits showed a decreasing pre-pandemic trend, and the decrease in the second quarter of 2020 itself did not differ significantly from this existing trend (B = −1.04, *P* = 0.052; Fig. 1). No significant change was observed for the number of telephone contacts in the second quarter of 2020.

**Fig. 2:**
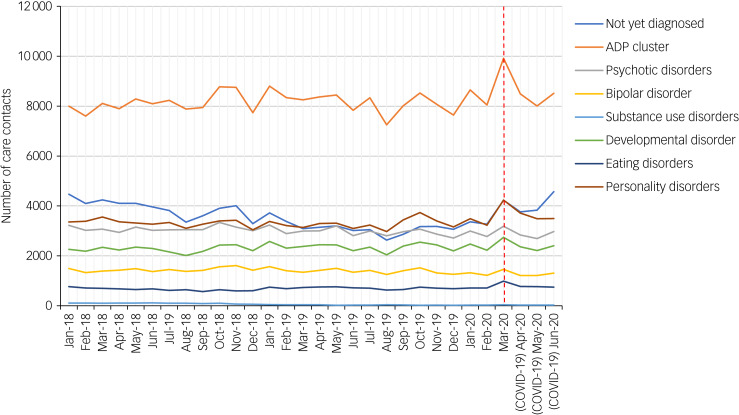
Number of care contacts by diagnostic category per month, from January 2018 to June 2020. All diagnostic categories show no significant changes after the lockdown announcement, as of 16 March 2020 (red dashed line). It should be noted that number of care contacts for all patients increases slightly a month before the lockdown, with patients from the ADP cluster showing relatively more elevation (orange line). ADP, anxiety–depression–post-traumatic stress disorder.

The absolute number of care contacts for each diagnostic category showed no changes in the second quarter of 2020 compared with the previous 2 years (Fig. 2). However, there were differences observed between diagnostic categories on the level of several communication modalities. In the second quarter of 2020, patients with psychotic disorders showed the least decrease in face-to-face out-patient contacts compared with other psychiatric diagnoses (Supplementary Fig. 1 available at https://doi.org/10.1192/bjo.2021.1049). Moreover, the same group showed relatively fewer video consultation frequency compared with other psychiatric diagnoses (Supplementary Fig. 2). We have to keep in mind that some of the diagnostic categories, such as eating disorders and substance misuse disorders, had relatively few patients (Table 1).

## Discussion

To our knowledge, this is the first study quantifying changes in mental healthcare utilisation, including telepsychiatry, during the first COVID-19 outbreak in continental Europe. We showed that the number of face-to-face out-patient contacts decreased as the number of video consultations increased, whereas the overall number of care contacts remained within the normal range of expected trend variation. This suggests that the reduced face-to-face out-patient contacts were fairly well offset by the increased implementation and widespread use of video consultations. Moreover, we show that the total number of care contacts remained the same for all diagnostic categories during the first COVID-19 outbreak. This implies that all patients received a similar amount of mental healthcare as before the pandemic. However, patients with psychotic disorders received relatively more face-to-face out-patient contacts and fewer video consultations compared with other psychiatric diagnoses during this period.

The changes in video consultations and face-to-face out-patient contact were comparable with the findings from Looi et al.^15^ Moreover, this report demonstrated that the combined number of face-to-face consultations and telepsychiatry contributed to a 14% increase in total mental health service use, whereas face-to-face consultations had declined during the second quarter of 2020 in Australia.^15^ The COVID-19 measures may have played an important role in this phenomenon, but there are several factors that may have also contributed. First, such an increase may reflect a growing demand for mental health services, as an exacerbation or relapse of pre-existing mental illness commonly occurs in the context of increased distress, such as the current pandemic.^21^ Anxiety, major depressive disorder and post-traumatic stress disorder are the most common presenting conditions that may occur together or alongside pre-existing disorders.^22^ This was probably the case for patients from the ADP cluster in the period immediately before the announcement of the lockdown, which shows an increase in absolute numbers. Second, the widespread implantation of telepsychiatry introduced new approaches to maintaining the patient–physician relationship. Especially in psychiatry, where patients have a long-term treatment relationship with their psychiatrist, these everyday conveniences, made possible by nowadays technology, allow flexibility in (re)scheduling appointments and create a sense of increased accessibility to mental healthcare. As a result, cancellations and no-show frequency may be reduced.^15^ Furthermore, technology can save time, cost and needed reliance on informal caregivers for commuting, thereby reducing the barriers to patients attending appointments. In addition, patients who experience a major barrier to being away from home because of disorder-specific issues could now opt for telepsychiatry, and thus continue routine meetings with the psychiatrist.^14^

However, patients who find it particularly difficult to use digital modalities or who lack socioeconomic characteristics to use them may feel even more inhibited in accessing mental health services.

### Patients with psychotic disorders and use of telepsychiatry

The association between diagnosis of a psychotic disorder and relatively less use of video consultations was in line with a pre-pandemic study reporting that psychotic disorders were more prevalent in the non-video consultation group compared with the video consultation group.^23^ Disorder-specific factors and attitudes of healthcare professionals may explain why changes for patients with psychotic disorders differ from those for other psychiatric disorders in regards to mental health service utilisation during the first COVID-19 outbreak. First, it may be that patients with psychotic disorders (or their caregivers) have a greater need for face-to-face contacts compared with patients with other psychiatric disorders. This could be related to the relative cognitive impairment associated with these disorders.^24–27^ In addition, symptoms such as paranoid delusions may render patients with psychotic disorders less likely to engage with digital modalities.^28,29^ Nonetheless, a number of pre-pandemic studies found that telepsychiatry has the potential of improving the quality of mental healthcare for these patients and their mental health condition, by promoting self-reliance.^29–33^ Second, stigma may play a role among healthcare professionals, as some may prefer face-to-face contact over virtual contact because of beliefs about the supposed difficulties that these vulnerable patients may encounter when using telepsychiatry.^29^ It could also be the case that health professionals feel negatively toward using digital modalities, out of concerns about possible detrimental effects on the therapeutic relationship.^29,34^ Furthermore, the current pandemic may magnify existing socioeconomic disparities and medical comorbidities, disproportionally predisposing patients with psychotic disorders to social isolation, and thus hindering accessibility to digital (mental) healthcare.^35,36^ On the other hand, it is possible for patients with severe mental illness to participate more actively in telepsychiatry when factors such as access to fast internet, technology and privacy-friendly living spaces are available.^37,38^ In The Netherlands, video consultation is a reasonably accessible option because >75% of the general population owns a smartphone; although little is known about such a percentage among patients with severe mental illness, it should be roughly similar.^39,40^ Since the passing of legislation in 2016 extending disability rights and providing protection against discrimination on the basis of disability or chronic illness, the Dutch Government has been promoting digital accessibility to healthcare. This, together with the timely announcement of a temporary subsidy scheme for digital healthcare, allowed face-to-face care contacts to be quickly converted to telepsychiatry at the beginning of the first COVID-19 outbreak.^41^

Strengths of this study include the size of our study population, the fact that psychiatric diagnoses were established by clinicians rather than from self-reports, and the time-series analyses that allowed us to capture and distinguish significant changes in several mental health services associated with the outbreak.

### Limitations

Nonetheless, our findings should be interpreted in light of several limitations. First, we were unable to assess or control for (associations with) socioeconomic and demographic factors because of Dutch privacy regulations. If possible covariates existed, we were unable to explore and adjust their potential effect on our findings. Second, for the sake of simplicity, we deliberately chose not to analyse or report data on types of mental health services (e.g. crisis contacts, intake of new patients, pharmacotherapy contacts, etc.). Third, we need to keep in mind that we could only present the main diagnostic categories that are reimbursed within our mental healthcare institute. An underreport of substance use disorder may be expected, given that substance use disorder as a primary disorder is not considered a diagnosis for which treatment is reimbursed. Alternatively, most patients with substance use disorders from the region Gelderland are treated at locally specialised centres, where related treatment reimbursement is possible.^42,43^ Fourth, we refrained from further analyses of differences between diagnostic categories within a communication modality, such as video consultations or face-to-face out-patient contacts. We believe that such detailed analyses could produce undefined results that would deviate from the objectives of this study. Instead, we have presented indices per diagnostic category that reflect the actual situation of both video consultations and face-to-face ambulatory contacts. Sixth, we clustered anxiety disorder, depressive disorder and post-traumatic stress disorder into one category. This may limit understanding of differences between these groups and their associations with mental health service utilisation. Seventh, we excluded in-patients and emergency contacts, which could have affected our finding of the relatively lower use of telepsychiatry in those with psychotic disorders if this specific group had been relatively more frequently admitted. However, recent studies of psychiatric emergency contacts show that regular out-patient contacts have decreased in all psychiatric patients since the pandemic, as a result of restricted face-to-face regulations, hinting at an non-selective decrease of admission rate for all psychiatric patients.^44–46^ Finally, our results cannot be generalised to all other mental healthcare settings because of inherent differences in mental health service provision across the globe.

In conclusion, the overall number of care contacts remained the same; however, telepsychiatry in general and video consultations in particular have increased substantially during the first COVID-19 outbreak in The Netherlands. In this period, psychotic disorders were associated with a relatively smaller increase in video consultations a smaller decrease in face-to-face contacts, relative to other disorders. Awareness of these associations among mental healthcare personnel may improve the quality of care for several vulnerable patient groups, by optimising access to the contemporary array of mental health services, including telepsychiatry. Future research should investigate whether optimisation indeed results in better care, and whether telepsychiatry is a relatively safe and reliable method for patients across the full range of mental illnesses. Moreover, future research should examine the impact of the pandemic on vulnerable groups such as youth and the elderly with mental illness, who may experience even more barriers to (digital) mental healthcare because of age-specific vulnerabilities, in addition to disorder-specific problems. Such insights into telepsychiatry could assist in optimising digital mental healthcare access in the post-pandemic era, and during possible future pandemics.

## Data Availability

The data that support the findings of this study are available from the corresponding author, M.W.C., upon reasonable request. The data are not publicly available due to Dutch privacy regulations.

## Supplementary material

For supplementary material accompanying this paper visit http://dx.doi.org/10.1192/bjo.2021.1049. click here to view supplementary material
