# Supplementary material for: Impact of the first COVID-19 outbreak on mental health service utilisation at a Dutch mental health centre: retrospective observational study
Source: BJPsych Open. 2021 Nov 17;7(6):e213. doi: 10.1192/bjo.2021.1049 (PMC8632375; doi:10.1192/bjo.2021.1049)
Supplement: Supplementary file 1 [file S2056472421010498sup001.docx]

**Supplementary Materials – Impact of the first COVID-19 outbreak and mental health service utilisation at a Dutch Mental Health Centre: retrospective observational study**

Man Wei Chow^1,2^, Eric Noorthoorn^3^, André Wierdsma^4^, Marte van der Horst^1,2,3^, Nini de Boer^1,2^, Sinan Guloksuz^5,6^, Jurjen J. Luykx^1,2,3^

^1^ Department of Psychiatry, UMC Utrecht Brain Centre, University Medical Centre Utrecht, Utrecht University, Utrecht, The Netherlands.

^2^ Department of Translational Neuroscience, UMC Utrecht Brain Centre, University Medical Centre Utrecht, Utrecht University, Utrecht, The Netherlands.

^3^ GGNet Mental Health, Warnsveld, The Netherlands

^4^ Department of Psychiatry, Erasmus Medical Centre, Rotterdam, The Netherlands

^5^ Department of Psychiatry and Neuropsychology, School for Mental Health and Neuroscience, Maastricht University Medical Centre, Maastricht, The Netherlands

^6^ Department of Psychiatry, Yale School of Medicine, 34 Park Street, New Haven, CT, USA

**Supplementary Fig 1.** Index number of face-to-face outpatient contacts per diagnostic category, from January 2018 to June 2020. Patients with psychotic disorders (orange line) showed the least decrease in face-to-face outpatient contacts compared to other psychiatric diagnoses after the lockdown announcement, as of 16-03-2020 (red dashed line).

**
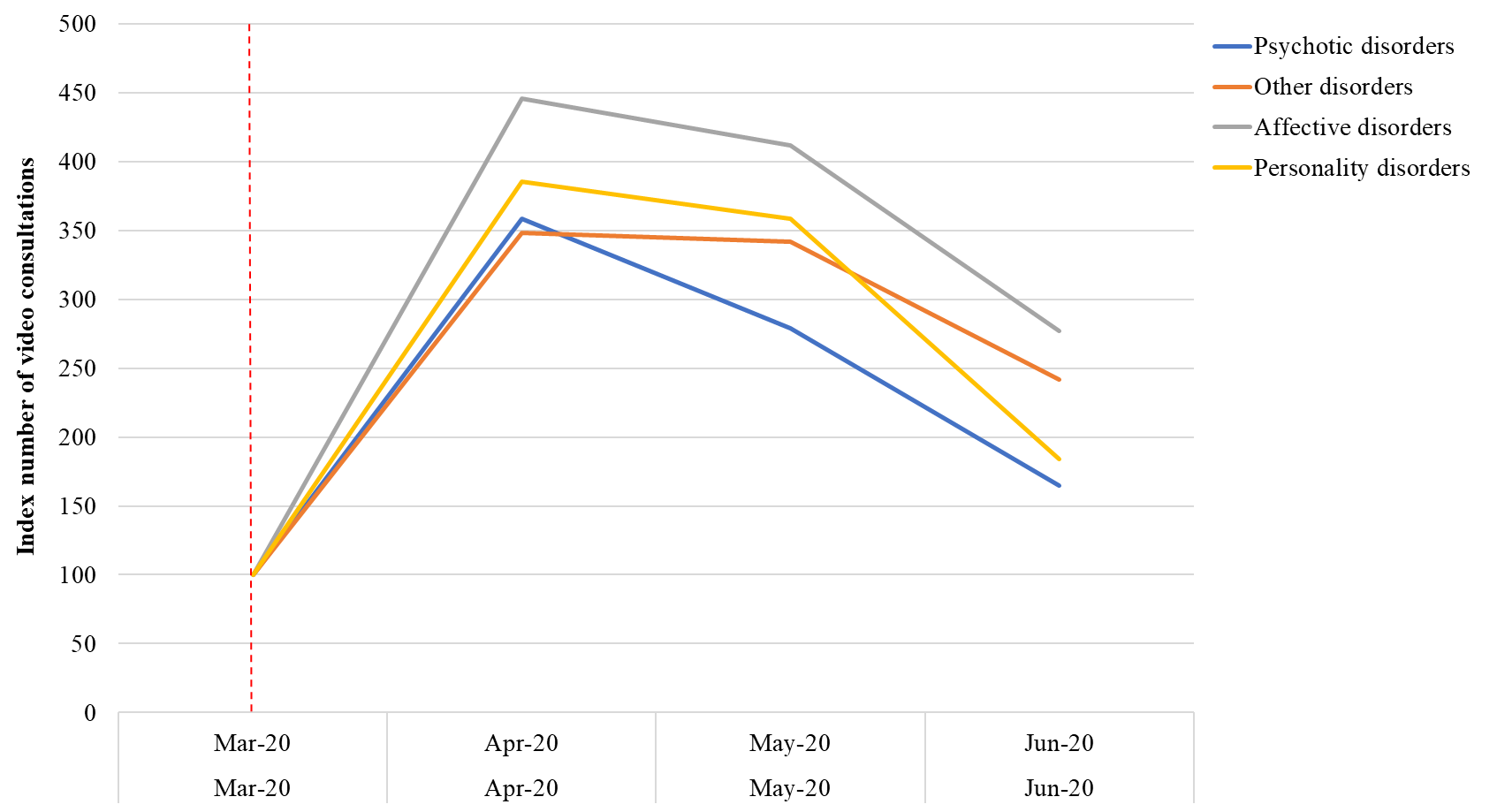
**

**Supplementary Fig 2.** Index number of video consultations per diagnostic category, from January 2018 to June 2020. Patients with psychotic disorders (blue line) show the least video consultation frequency compared to other psychiatric diagnoses after the lockdown announcement, as of 16-03-2020 (red dashed line). Some diagnostic categories are aggregated together due to relatively small number of video consultations: other disorders consist of developmental disorders, eating disorders, substance use disorders, and the not yet diagnosed group; Affective disorders consist of anxiety disorders, depressive disorders, PTSD, and bipolar disorders.
